# Mechanism of cognitive processing for acupuncture action on generalized anxiety with naturally occurring consecutive partial sleep deprivation in early adulthood: a randomized controlled study and evaluation of event-related potentials

**DOI:** 10.3389/fpubh.2024.1420299

**Published:** 2024-10-16

**Authors:** Ce Shi, Lihua Wu, Wen Fu, Jing Gao, Haishui Jiang, Mengyu Wang, Xinwang Chen

**Affiliations:** ^1^College of Acupuncture, Moxibustion and Tuina, Henan University of Chinese Medicine, Zhengzhou, China; ^2^Rehabilitation Medicine College Henan University of Chinese Medicine, Zhengzhou, China; ^3^Department of Rheumatology, The First Affiliated Hospital of Henan University of Chinese Medicine, Zhengzhou, China; ^4^Department of Rehabilitation Center, The First Affiliated Hospital of Henan University of Chinese Medicine, Zhengzhou, China

**Keywords:** generalized anxiety disorder, partial sleep deprivation, acupuncture, event-related potentials, cognitive function, early adulthood

## Abstract

**Introduction:**

Generalized anxiety disorder (GAD) is a common mental disorder that often begins in adolescence or early adulthood and is characterized by widespread and persistent anxiety. Partial sleep deprivation (PSD) is an important risk factor for GAD development and a common comorbidity. Adolescence is a period of rapid brain and nervous system development, and during this time, the occurrence of GAD can lead to neurocognitive deficits, such as impaired attention, cognitive control, and attention bias, that significantly affect cognitive function. However, relatively little research has been conducted on GAD comorbid with PSD in early adulthood compared with other psychiatric disorders. Clinical studies have demonstrated the effectiveness of acupuncture in treating GAD and sleep disorders, but the mechanism of how acupuncture modulates neurocognitive processing in patients with GAD comorbid with PSD has not been clarified.

**Methods/design:**

In this randomized clinical trial, a total of 56 participants diagnosed with GAD comorbid with naturally occurring PSD and 28 healthy controls (HCs) will be recruited. The participants diagnosed with GAD comorbid with PSD will be randomly assigned to either the acupuncture group or the sham acupuncture group at a 1:1 ratio. The primary outcome measure is the Hamilton Anxiety Rating Scale (HAMA). Secondary outcome measures are the Sleep Deprivation Index (SDI), the Self-Assessment Scale for Anxiety (SAS), the Epworth Sleepiness Scale (ESS), and the State-Trait Anxiety Inventory (STAI). Additionally, three psychological paradigms (the attentional network test, psychomotor vigilance test, and emotional face Go/No-go) and event-related potential (ERP) data. Healthy volunteers will not undergo acupuncture but will instead participate in baseline assessments for the scales, mental paradigms, and ERP data. Acupuncture and sham acupuncture interventions will be conducted for 30 min, three times a week, over a 2-week period. Evaluations will be performed at zero weeks (baseline), 1 week, and 2 weeks, with the data enumerator, outcome assessor, and participant blinded to the treatment assignment.

**Discussion:**

This study contributes to the exploration of the effects of acupuncture on improving anxiety symptoms and cognitive functions in individuals with comorbid GAD and PSD.

**Trial registration:**

ClinicalTrials.gov, ChiCTR2400082221. Registered March 25, 2024.

## 1 Introduction

### 1.1 Partial sleep deprivation leads to anxiety disorders in early adulthood

The National Sleep Foundation defines partial sleep deprivation (PSD) in early adulthood as sleeping < 6 h (1). Sleep and mood are closely related, and studies have shown that sleep deprivation is associated with increased physiological arousal, decreased ability to experience positive emotions, and increased sensitivity to negative emotional experiences (2). Sleep disorders are a major predictor of anxiety attacks (3), and they are also responsible for the onset of anxiety symptoms and the worsening of baseline anxiety states. A study of sleep conditions and anxiety states showed that naturally occurring partial sleep deprivation in school students led to increased levels of self-reported anxious arousal symptoms the next day (4). Naturalistic studies of the relationship between sleep and mood experiences in non-clinical samples have also shown that shorter sleep duration is typically associated with increased negative mood and anxiety and depressive symptoms the next day (5).

Generalized anxiety disorder (GAD) is characterized by widespread and persistent anxiety, accompanied by palpitations, sweating, tremors, dry mouth, sleep disturbances, irritability, poor concentration, and muscle tension, often starting in adolescence or early adulthood. GAD is often comorbid with other types of anxiety disorders and mood disorders, with sleep disorders or insomnia being one of the most predominant comorbidities (6). Sleep disorders are very common in individuals experiencing anxiety and are a defining criterion for many anxiety or anxiety-related disorders (7). Individuals suffering from anxiety often exhibit sleep disturbances, particularly sleep deprivation (8), that can trigger or further exacerbate anxiety symptoms (9). Psychobiological models suggest that a state of hyperarousal is a key factor in insomnia (10–12). Insomnia may be due to excessive cognitive dysfunction, maladaptive behaviors, physiological hyperarousal, and mental hyperarousal. Individuals suffering from insomnia often worry about sleep deprivation and the consequences of sleep deprivation; therefore, their anxiety-related beliefs, attitudes, and behaviors contribute to the persistence or worsening of sleep disturbances and severely shorten sleep duration (13). In summary, sleep deprivation is an important risk factor for generalized anxiety disorder and its common comorbidity, and there is an interactive relationship between the two.

### 1.2 Comorbidity of GAD and PSD is associated with cognitive dysfunction

It has been found that patients with anxiety disorders often have unconscious emotional processing deficits, working memory deficits, and attention and vigilance deficits. In addition, negative cognitive processing bias is one of the most important causes of the emergence, persistence, and development of anxiety (5). GAD in early adulthood is often accompanied by varying degrees of neurocognitive dysfunction that affects the ability to learn and remember, make behavioral decisions, and perceive and comprehend feelings. These lead to impairments in executive functioning, attention, memory, and information processing speed (14). These may manifest as procrastination, lack of confidence, indecisiveness, poor decision-making, distractibility, and memory loss, but the specific manifestations vary from person to person (15). Adolescence is a time of rapid brain development, and naturally occurring PSD during adolescence or early adulthood can lead to cognitive declines in learning and memory (16). Some studies have concluded (17) that insomnia in adolescents can affect their cognitive deficits directly or indirectly through the chain-mediated effects of fatigue, depression, and anxiety. In a study of college students, participants in the sleep deprivation group were deprived of sleep for 24 h, whereas participants in the non-sleep deprivation group were allowed to sleep in their beds for ~8 h under normal sleep conditions. Afterward, all participants completed cognitive tasks. Participants also provided self-assessment reports on their mood, effort, attention, performance estimates, and off-task cognition (18). Results indicated that participants in the sleep deprivation group performed significantly worse on the cognitive tasks compared with the non-sleep deprivation group. However, participants in the sleep deprivation group rated their attention, effort, and performance estimates higher than participants in the non-sleep deprivation group. This study suggests that college students may not be fully aware of the negative effects of sleep deprivation on their cognitive abilities. Investigating the neurobiological mechanisms underlying the comorbidity of GAD and early adult sleep disorders may help improve cognitive functioning in patients with GAD and may have important implications for the diagnosis, prevention, and treatment of GAD and related psychiatric disorders.

### 1.3 Acupuncture has favorable clinical efficacy in treating GAD and sleep disorders

Currently, the treatment of GAD involves medication and psychotherapy. Psychotherapy, while effective, typically lasts longer, requires a long-term commitment, and may not produce sustained symptom relief (19). If symptoms do not improve or only partially improve after a certain period of treatment, medication may be required. Medication primarily involves the use of benzodiazepines, 5-HT1A receptor partial agonists, and antidepressants with anxiolytic effects, such as SSRIs and SNRIs. Medication has been associated with a variety of side effects, including, but not limited to, addiction, medication tolerance, decreased attention to novel stimuli, memory loss, dizziness, nausea, diarrhea, decompensation, and weight gain (20). Currently, improvement rates with first-line treatment medications are typically 60%−70%. However, residual symptoms remain in 30%−60% of cases (21).

Acupuncture is an effective, safe, intervention with low side effects. Clinical studies have shown that acupuncture can significantly improve sleep quality and cognitive function in individuals with sleep deprivation (22) and rapidly reduce anxiety levels in patients with anxiety (23). Brain function research confirms that patients with anxiety and sleep disorders exhibit deficiencies in the default mode network. Repetitive negative thinking caused by poor sleep quality inhibits default mode network activation and internal functional connectivity, thereby reducing the ability to inhibit and regulate anxiety symptoms (24). There is a high degree of overlap between brain regions responsive to acupuncture and those within the default mode network (25). Research indicates that acupuncture can modulate functional connectivity within the default mode network (26–30), and increasing the number of acupuncture points or the intensity of stimulation can enhance regulation of the default mode network (31). However, the clinical efficacy and influence on cognitive processing pattern of acupuncture in patients with GAD comorbid with PSD remain unclear. Given this, the present study uses the General Anxiety Disorder-7 (GAD-7), the Self-Rating Anxiety Scale (SAS), the Hamilton Anxiety Rating Scale (HAMA), the State-Trait Anxiety Inventory (STAI) anxiety scales, the Sleep Deprivation Index (SDI), and the Epworth Sleepiness Scale (ESS) sleep scales to observe the clinical efficacy of acupuncture on patients with GAD comorbid with PSD. In addition, the event-related potentials (ERPs) in combination with the Go/No-go task, the attentional network test (ANT), and the psychomotor vigilance test (PVT) are used to observe the neurocognitive characteristics of PSD patients and the electrophysiological mechanism of acupuncture that affects the comorbidities of GAD and naturally occurring PSD to provide experimental evidence for the prevention and treatment of PSD in early adulthood.

### 1.4 Objectives

This study aims to observe participants with comorbid GAD and PSD to explore the intrinsic brain cognitive mechanisms associated with naturally occurring PSD in conjunction with GAD. Furthermore, the study seeks to investigate the clinical effects of acupuncture on improving both sleep deprivation and GAD symptoms in this comorbid population.

## 2 Methods/design

### 2.1 Study setting

**Figure 1:**
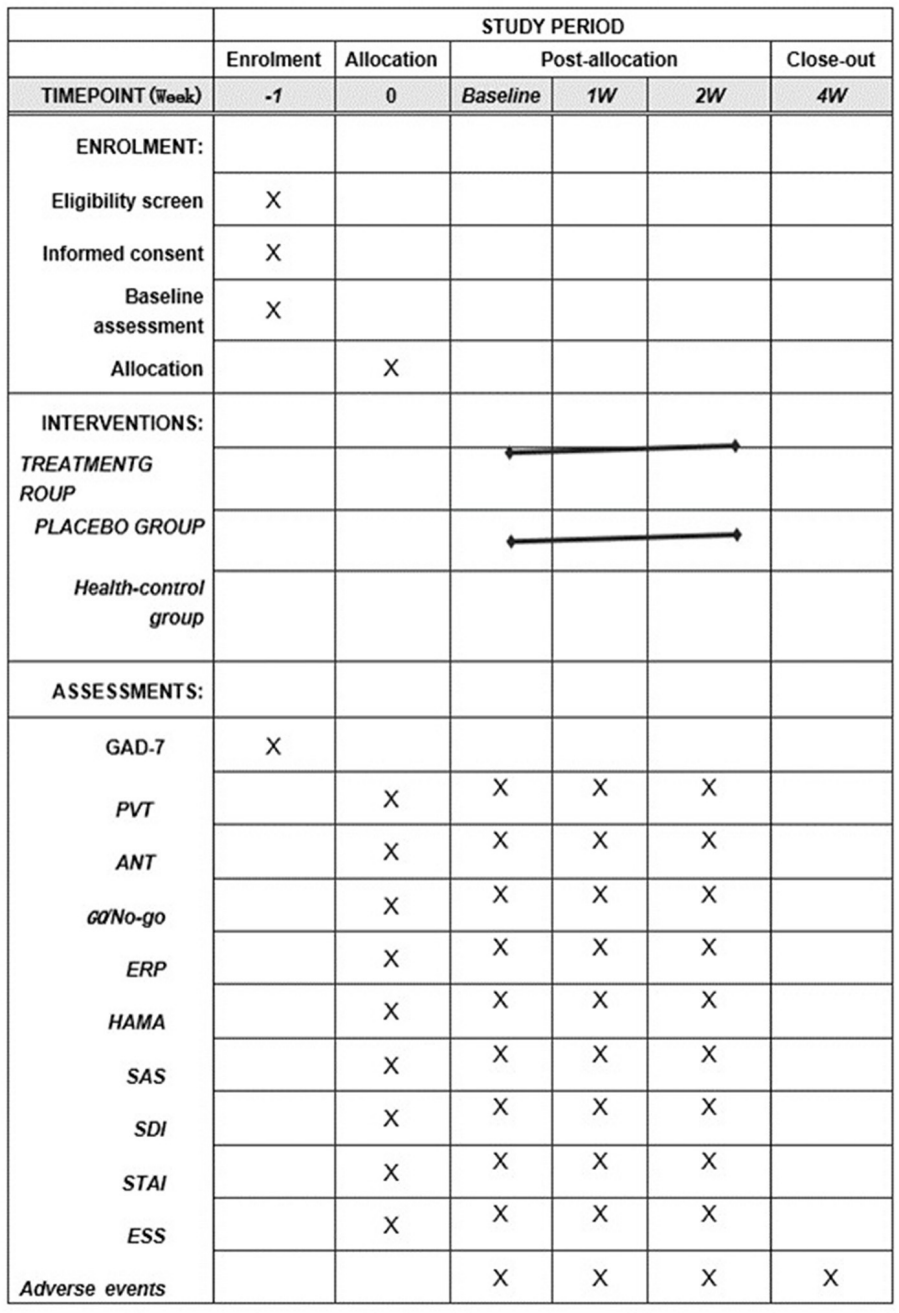
SPIRIT table of the schedule of enrollment, interventions, and assessments. PVT, Psychomotor Vigilance Test; ANT, Attention Network Test; ERPs, event-related potentials; HAMA, Hamilton Anxiety Rating Scale; GAD-7, General Anxiety Disorder-7; SAS, Self-Rating Anxiety Scale; SDI, Sleep Deprivation Index; STAI, State-Trait Anxiety Inventory; ESS, Epworth Sleepiness Scale; and GO/No-go, Go/No-go Emotional Detection Paradigm.

This study will be a parallel randomized controlled clinical trial with the participants blinded to their groups. The research will be conducted in accordance with the principles outlined in the Consolidated Standards of Reporting Trials (CONSORT) and the Standards for Reporting Interventions in Clinical Trials of Acupuncture (STRICTA). A total of 56 participants diagnosed with GAD comorbid with naturally occurring PSD (i.e., nighttime sleep duration ≤ 6 h) will be recruited, and 28 healthy volunteers will also be recruited as a control group. Participants will be randomly assigned at a 1:1 ratio to either the acupuncture group or the sham acupuncture group. The establishment of the sham acupuncture group is intended to provide clearer evidence of the therapeutic effects of acupuncture and to compare these effects with the placebo effect of sham acupuncture. Both the acupuncture and sham acupuncture interventions will be conducted over a 2-week period, with sessions conducted three times a week. The study will be conducted at the Third Affiliated Hospital of Henan University of Chinese Medicine in Henan Province, China. The research will follow the Standard Protocol Items: Recommendations for Interventional Trials (SPIRIT) guidelines (32). For the participant timeline, see Figure 1.

### 2.2 Recruitment

The study will enlist participants with GAD and comorbid naturally occurring PSD (nightly sleep duration ≤ 6 h) from Longzihu University City and the East Minglu Campus of Henan University of Chinese Medicine through poster placement and online recruitment. Participants who meet the inclusion criteria, do not meet any exclusion criteria, and agree to participate in this trial will be included, and they will sign an informed consent form.

### 2.3 Inclusion criteria

The inclusion criteria are as follows:

1) Between 18 and 25 years of age.
2) An average sleep duration equal to or < 6 h per night in the past 30 days.
3) A score of ≥5 points on the 7-item Generalized Anxiety Disorder Scale (GAD-7).
4) No anti-anxiety or insomnia-related medications taken or acupuncture treatments for anxiety or insomnia received in the most recent 30 days.
5) Willingness to participate in the clinical study and sign an informed consent form.

### 2.4 Exclusion criteria

Participants with any of the following conditions will be excluded:

1) Organic mental disorders, anxiety, or depression related to surgical history or psychoactive substances.
2) Family history of other mental illnesses, such as dementia, schizophrenia, mania, or addiction.
3) Cognitive impairments that make subjects unable to cooperate.
4) Pregnancy, preparing for pregnancy, or breastfeeding.
5) Skin lesions or skin diseases, severe diabetes, tumors, or severe cardiovascular, lung, liver, kidney, and hematopoietic system diseases.
6) Patients with contraindications to electroencephalography, as well as those with encephalitis, cerebrovascular diseases, or intracranial space-occupying lesions that affect the interpretation of EEG results.

### 2.5 Dropout criteria

Included subjects will be dropped from the study in the following cases:

1) Subjects with poor compliance who quit on their own during the treatment.
2) Those who use treatment methods prohibited by this plan or change the treatment method on their own.
3) Cases where serious adverse reactions or complications occur and are not suitable for continued treatment and the trial is terminated.

Dropped cases will be handled as follows:

1) After the subject drops out, the attending doctor will try to contact the subject to ask for the reason by visiting them after making an appointment by phone or letter, and they will then record the last treatment time and complete the assessable items.
2) For cases that quit the trial due to adverse reactions or ineffective treatment, the attending doctor will take appropriate measures according to the actual situation of the subjects.
3) The “Summary of Treatment Completion” and “Clinical Trial Completion” in the CRF will be filled in.
4) All excluded and dropped cases will be subjected to intention-to-treat analysis after the trial ends.

### 2.6 Randomization

Using a simple randomization method, we will input the estimated sample size into the SPSS 18.0 statistical software package, generate random seeds, generate random numbers, obtain the permutation order to get the grouping results, make random cards, and then place them into opaque envelopes and seal them. During clinical implementation, the order of qualified cases included in the trial corresponds to the order of the numbers on the envelopes. We will open the envelopes in this order and group subjects according to the prompts on the randomized cards.

### 2.7 Blinding

We will implement a three-way separation of efficacy evaluators, acupuncture operators, and statisticians. Because the control group uses sham acupuncture, it is possible to blind the subjects. Blinding will be assessed after the last treatment using a questionnaire that asks participants if they were in the real treatment group or the sham treatment group, with possible responses of “real treatment group,” “sham group,” or “do not know.”

### 2.8 Intervention

All practitioners in this trial are licensed traditional Chinese medicine acupuncture therapists with at least 5 years of clinical experience, and they will be trained to master the study protocol. The acupuncturist will be asked to administer the standard manipulation for treatment.

### 2.9 Treatment group

The acupuncture group acupoints used will be Baihui, Yintang, Hegu, Taichong, Neiguan, and Shenmen. After routine skin disinfection, the adhesive pad and plastic guide base of the Park acupuncture needle device will be attached to the skin surface. An acupuncture needle with a diameter of 0.35 mm and a length of 25 mm will be placed inside the plastic guide, and it will be inserted into the skin along the direction of the guide. There will be no lifting or twisting manipulations performed after the needle is inserted into the acupuncture point. The selection of acupuncture points will follow the standards outlined in the National Standard of the People's Republic of China, titled “Names and Locations of Acupoints” (GB/T 12346-2006).

### 2.10 Sham acupuncture group

The acupoints for the sham group will be the same as those in the acupuncture group. After routine skin disinfection, the adhesive pad and plastic guide base of the Park acupuncture needle device will be attached to the skin surface. An acupuncture needle with a diameter of 0.40 mm and a length of 25 mm will be placed inside the plastic guide. Light tapping or pressure techniques will be applied without penetrating the skin.

### 2.11 Health control group

**Figure 2:**
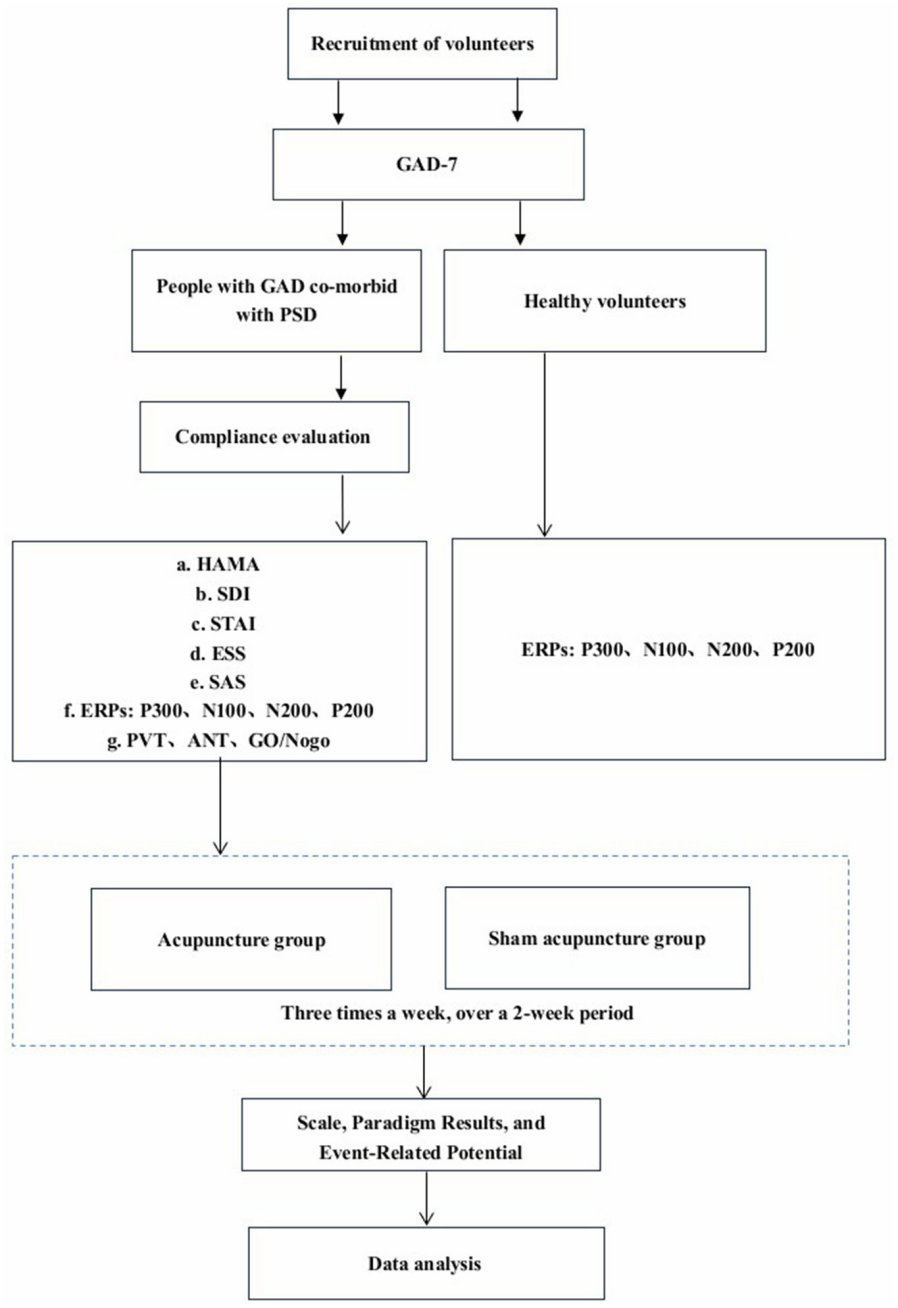
Trial work plan. The main outcome measure is HAMA. The remaining outcome measures are the secondary outcomes of the study.

Healthy controls and patients were included at a 1:2 ratio. The healthy control group will not receive any intervention. They will maintain normal work and rest times. During the baseline period, the same scales and cognitive function assessments were conducted as those performed on the patients. For the trial work plan, see Figure 2.

### 2.12 Outcomes

Since the HAMA is essentially related to the purpose of the study, it is an observation index that can accurately reflect the effectiveness of acupuncture and is a recognized evaluation standard in anxiety research. Therefore, this trial adopted the HAMA as the primary outcome indicator. In order to evaluate the degree of anxiety and symptoms of the participants from multiple dimensions and analyze the differences between subjective and objective assessments, this study added secondary outcome indicators. When the primary outcome indicator has statistical significance, the statistical analysis results of the secondary outcome indicators are of reference value.

#### 2.12.1 Primary outcome

The primary outcome measure will be the Hamilton Anxiety Rating Scale (HAMA). Developed by psychiatrist Max Hamilton in 1959, HAMA aims to provide a quantified and standardized method for assessing anxiety symptoms (33). It was one of the first scales in the field of mental health and continues to be widely utilized. HAMA consists of 14 items, each targeting different anxiety symptoms or signs. These items include emotional anxiety, somatic anxiety, insomnia, intellectual functioning, somatic symptoms, behavioral symptoms, fear of illness, psychological symptoms, reproductive symptoms, gastrointestinal symptoms, respiratory symptoms, cardiovascular symptoms, muscle symptoms, and sensory organ symptoms. Scores for each item range from 0 (no symptoms) to 4 (severe symptoms). The total score ranges from 0 to 56 points. Higher scores indicate more severe anxiety symptoms. A score > 17 indicates mild anxiety, while scores between 25 and 30 are considered moderate to severe anxiety (34). HAMA is used not only in clinical assessments, but also in research settings to evaluate the effectiveness of drug treatments, to monitor changes in anxiety symptoms, or to serve as a diagnostic tool. HAMA is thus a comprehensive and complex tool for assessing and monitoring anxiety symptoms in adults. The reliability and validity of HAMA are adequate (Cronbach's alpha is 0.921, and validity is 0.529–0.727) (35, 36). With wide applications in both clinical and research environments, it has become one of the standard tools in the field of mental health.

#### 2.12.2 Secondary outcomes

The Sleep Deprivation Index (SDI) is used to assess the extent of sleep deprivation in patients. It is a useful tool for quantifying and evaluating a person's level of sleep deprivation. The SDI primarily collects data on the difference between average weekend sleep duration and average weekday sleep duration, represented numerically (37). The content of the SDI includes responses to the following questions: (a) In the past 2 weeks, how many hours did you sleep on average on weekdays? b) On weekends, if no one wakes you up, how many hours do you sleep on average? By collecting data on the Sleep Deprivation Index, the degree of sleep deprivation in participants can be observed to assess its correlation with anxiety status.

The Epworth Sleepiness Scale (ESS) is used to assess daytime sleepiness. The ESS is a widely used questionnaire developed by Australian sleep expert Dr. Murray Johns in 1991 (38). The ESS consists of eight items, each describing typical situations in daily life and requiring respondents to assess the likelihood of falling asleep in these situations. Each item is scored on a range from 0 to 3 points: 0, never doze; 1, slight chance of dozing; 2, moderate chance of dozing; 3, high chance of dozing. The total score ranges from 0 to 24, with higher scores indicating greater daytime sleepiness. The interpretation of total scores is as follows: 0–5, normal daytime alertness; 6–10, normal range of sleepiness; 11–12, mild sleepiness; 13–15, moderate sleepiness; 16–24, severe sleepiness (38). The ESS is mainly used for the diagnosis and assessment of disorders related to excessive daytime sleepiness, such as obstructive sleep apnea and narcolepsy. It can also be used to assess the effectiveness of treatments or as a research tool. The ESS's internal consistency is generally considered good, with Cronbach's alpha coefficients typically ranging from 0.7 to 0.9. Some studies have also found that the ESS has good test–retest reliability, indicating relative consistency in scores for the same respondent at different time points (39). The validity of the ESS is widely supported, as its items are based on common daily activities directly related to daytime sleepiness. The correlation between ESS scores and other measures of sleep quality and sleepiness (such as polysomnography) has been confirmed (39).

The State-Trait Anxiety Inventory (STAI) is a psychological measurement tool used to assess an individual's level of anxiety. Developed by American psychologist Charles Spielberger and his colleagues in the 1970s, it is widely used in clinical and research settings (40). The STAI consists of two independent scales, measuring state anxiety and trait anxiety. State anxiety refers to the level of anxiety an individual experiences at a specific moment or in a particular situation. It is a transient emotional experience related to a specific context. The state anxiety portion of the STAI includes 20 items that ask participants to describe their current feelings. Trait anxiety refers to an individual's general and stable tendency toward anxiety, reflecting their overall reaction to threats and dangers. The trait anxiety portion of the STAI also consists of 20 items, asking participants to describe their usual feelings. Each item on the STAI is scored on a four-point scale, ranging from “almost never” to “very much,” with varying degrees of consistency. The total score for each scale ranges from 20 to 80, with higher scores indicating a higher level of anxiety. The STAI can be used for individuals of different ages and backgrounds, including children, adolescents, and adults. It has widespread applications in clinical assessment, research, and educational environments, used for diagnosing anxiety disorders, evaluating treatment outcomes, and studying the relationship between anxiety and other variables (41). The internal consistency reliability of the STAI is typically high, with Cronbach's alpha values ranging from 0.86 to 0.95, indicating high consistency among questionnaire items. The test–retest reliability of the STAI has also been demonstrated to be reliable. Some studies have found that the test–retest reliability of the Trait Anxiety Scale ranges from 0.65 to 0.75, while that of the State Anxiety Scale ranges from 0.16 to 0.62. This suggests that the Trait Anxiety Scale has higher stability, while the State Anxiety Scale is more influenced by specific situations. The content validity of the STAI is widely recognized, as it covers multiple aspects of anxiety, including physiological, cognitive, and emotional components. Through factor analysis, researchers have demonstrated that the two STAI subscales (state and trait anxiety) are independent, supporting its structural validity (41). The STAI shows a high correlation with other anxiety measurement tools, such as the Taylor Manifest Anxiety Scale, further validating its standard effectiveness (42).

The Self-Rating Anxiety Scale (SAS) is a self-assessment tool designed to evaluate an individual's level of anxiety, with the aim of quantitatively assessing the patient's anxiety status in a clinical setting (43). The SAS employs a four-point scoring system, primarily assessing the frequency of symptom occurrence. Among the 20 items, 15 are framed with negative wording and scored on a scale from 1 to 4 as described above. The remaining five items (5, 9, 13, 17, 19) are presented with positive wording and scored in reverse order from 4 to 1. According to the standard scores based on Chinese norms, the cutoff values for SAS are set at 50 points, with scores ranging from 50 to 59 indicating mild anxiety, 60–69 indicating moderate anxiety, and scores above 70 indicating severe anxiety. Raw scale scores for the SAS range from 20 to 80, with satisfactory psychometric characteristics that include internal consistency (Cronbach's alpha = 0.82) and concurrent validity (*r* = 0.30 with the Taylor Manifest Anxiety Scale) (43, 44).

Event-Related Potentials (ERPs) will also be used as a secondary outcome. The study will employ the PVT, ANT, and Go/No-go emotional detection paradigm to observe differences in the P300, N100, N200, P200, and other EEG changes related to participants' psychomotor vigilance, attention network effects, and emotional attention bias.

The PVT is a neuropsychological test designed to measure an individual's vigilance and reaction time (45). It is widely used in research on and the assessment of cognitive function changes related to sleep deprivation, work stress, medications, and other factors. The PVT is typically a simple visual reaction time task where participants need to respond as quickly as possible to randomly presented visual stimuli, often a numerical timer starting from 0. The time from stimulus onset to the participant pressing a button is recorded as reaction time. The PVT can be used to measure various parameters, including average reaction time, fastest and slowest reaction time, standard deviation of reaction time, as well as errors, such as premature button presses or failures to respond. The PVT is an extensively used tool for measuring vigilance and reaction time, capturing the impact of various factors on cognitive function through a straightforward visual reaction task, providing a detailed assessment of participants' attention. PVT measures sustained attention and is used to assess the effect of drowsiness on brain operational efficiency. PVT performance is considered to reflect a person's arousal and attention state and is highly sensitive to drowsiness and sleep deprivation. When sleep deprivation occurs, the amplitude of the P300 wave decreases, and its latency increases. It has also been shown that the P300 latency increases with pre-sleep drowsiness (46). Research has demonstrated that PVT is an effective and reliable indicator that can quantify alertness changes related to sleep restriction over a period of 3 months (47).

The ANT is a cognitive test used to measure and evaluate the functionality of attention networks. Developed by Michael I. Posner and other researchers, the ANT is designed to study how people control attention and process information. The ANT combines measurements of different types of attention, including alerting, orienting, and executive control (48). The ANT is based on three main attention networks: alerting network, orienting network, and executive control network. It typically consists of a series of rapidly presented visual stimuli, requiring participants to respond as quickly and accurately as possible. The key components of the test include central arrows, peripheral arrows, and cue stimuli. By analyzing participants' reaction time and accuracy, the ANT can measure parameters such as alerting effects, orienting effects, and conflict effects. The ANT is a tool for measuring and understanding various aspects of attention and finds widespread applications in cognitive science, clinical psychology, developmental psychology, and other fields (49). Patients with anxiety often exhibit symptoms of decreased attention. Various prolonged workloads can deplete attentional resources. ERP studies on sustained attention and vigilance contribute to understanding the brain mechanisms that support and maintain prolonged attention and vigilance (50). The research on mental fatigue using various tasks generally shows that with extended performance, the amplitudes of multiple ERP components, including N100 and P300, decrease (51). In studies of sustained attention, N100 is associated with the early stages of selective attention to task stimuli, while P300 is an indicator of the later stages of conscious stimulus evaluation and classification (52). Relevant research indicates that the Attention Network Test (ANT) demonstrates high reliability in execution in clinical use but low reliability in alerting and orienting scores (48, 53).

In Go/No-go emotional detection, emotional attention bias refers to an individual's tendency when processing emotional information, especially when emotional information competes for attention with other non-emotional information (54). The Go/No-go emotional detection paradigm is an experimental method used to study emotional attention bias, and related ERPs provide insight into how the brain processes this information (55). In the Go/No-go task, participants are instructed to respond to specific types of stimuli (e.g., speech or faces with specific emotions) and withhold responses to other types of stimuli. This paradigm can be used to study attention and response control to different emotional stimuli. Patients with anxiety often exhibit impaired cognitive control and avoidance behaviors. Inhibitory control is an important component of the cognitive control system, and studies suggest that the inability to inhibit responses to negative stimuli is related to anxiety. The N2 component in emotion-related GO/No-go tasks is associated with conflict monitoring processes, while the P3 component is more related to conflict resolution and behavioral inhibition (56). In a previous study that involved an emotion-related GO/No-go task, anxious patients showed reduced amplitude differences at the N2 stage when required to inhibit facial stimuli. The GO/No-go amplitude for neutral faces predicted self-reported anxiety levels (57). These results suggest that dysfunctional inhibitory control may be a characteristic marker of anxiety related to a decreased conflict monitoring ability or decreased active inhibition capacity.

### 2.13 Adverse events

Common treatment-related adverse events include acupuncture breakage, fainting during needling, local hematoma, infection, and abscess, though other discomforts can also occur after acupuncture (referring to post-acupuncture symptoms such as post-needling pain, nausea, vomiting, palpitations, dizziness, headache, anorexia, and insomnia). During the implementation of the project, specific record forms will be created, and patients will be asked whether any of the above situations have occurred during each diagnosis and treatment session. If so, these situations will be recorded immediately. Adverse event data will include the occurrence, duration, and severity of adverse reactions (symptoms and signs), as well as how the events were resolved (or unresolved) during treatment. Any adverse events occurring in subjects during the trial, including laboratory test abnormalities, will be carefully inquired about and investigated. Abnormal laboratory values or examination results are only considered adverse events when they cause clinical symptoms or signs, are considered clinically significant, or require treatment. All adverse events will be assessed in terms of their nature, severity, and correlation with medication and strictly recorded in the case report form. In the event of a serious adverse reaction, the team leader's unit and the reporting unit will be notified within 24 h, and a serious adverse event form will be filled out in this case. The handling of serious adverse events is reported, managed, and followed up on according to Good Clinical Practice principles. Handling methods may include one or more of the following: taking no action (i.e., further observation only); adjusting/temporarily suspending research treatment; permanently stopping research treatment due to the adverse event; adding concomitant drug treatment; administering non-drug treatment; admitting the patient to the hospital; or extending the patient's hospital stay. The measures taken to handle adverse events will be recorded on the adverse event page of the CRF.

### 2.14 Follow-up

Follow-up will take place 2 weeks after completion of the treatment program. This time point was selected to assess the sustained long-term effectiveness of the intervention.

### 2.15 Sample size

According to the results of a pilot study (58), after 8 weeks of treatment, the HAMA scores were 9.00 ± 3.2 in the treatment group and 10.83 ± 3.06 in the control group. Sample size estimation using a two-tailed test for a study with a paired design for two-sample mean comparison, a significance level of α = 0.05 (two-tailed), and a power of 1–β = 0.90 was performed. Using PASS 15.0 to estimate the difference between the two groups' HAMA as 0.915, assuming a common standard deviation of 1.83, the sample size was estimated using a two-sample test assuming equal variances in PASS 15.0. The estimation formula (60) is:


$$\begin{array}{l} n=\frac{2\times {\left(u_{\alpha }\;+\;u_{\beta }\right)}^{2}\sigma^{2}}{\delta } \end{array}$$

Considering ~20% potential dropout during the observation period, the calculated sample size was calculated to be ~28 cases per group, and the total number of participants was determined to be 56.

### 2.16 Data management and monitoring

The case report form (CRF) includes observation time points, outcome measures, adverse events, and safety evaluations. The outcome assessors will be required to follow the requirements of the CRF and fill in the relevant information in a timely and accurate manner. Both paper documents and electronic records will be retained for at least 5 years after publication. In case of inquiries, readers are encouraged to reach out to the corresponding author for access to the original data. Protection of patient confidentiality is of paramount importance, encompassing details such as name, ID number, and telephone number. The research protocol will undergo scrutiny and refinement by experts in acupuncture, emergency procedures, methodology, and statistics. Adhering to a predetermined standard operating procedure, our approach incorporates patient screening, enhancement of relevant inspections, acupuncture administration, completion of CRFs, outcome assessments, and meticulous data management. Outcome assessments, CRF completion, and data management are maintained under rigorous supervision. The electronic data will be stored in the ResMan Research Manager repository. Anonymized trial data will be shared with other researchers at least 3 years after the article is published. If reviewers or readers have any questions regarding our published data, they can contact the corresponding author for access to the original data or visit ResMan (http://www.medresman.org/uc/project/projectadd.aspx). The Institutional Ethics Committee (IEC) at the Third Affiliated Hospital of Henan University of Chinese Medicine will oversee our activities independently of investigators and sponsors, conducting audits of trial conduct on a 12-month basis. Any alterations or corrections to operational procedures will be comprehensively documented through a breach report form, subject to monitoring, and submitted to the directors of the ethics committee and the China Clinical Trial Registration.

### 2.17 Data analysis

Data analysis will include case distribution, comparability analysis, efficacy analysis, analysis of factors affecting efficacy, and safety analysis. Statistical analyses will be conducted using PASS 18.0 statistical analysis software. First, a baseline analysis of the demographic characteristics of the cases in both groups will be conducted to assess the balance and comparability between the two groups. Subsequently, the efficacy and safety indicators of the two groups will be compared. For intra-group comparisons of quantitative data between the experimental and control groups, paired *t*-tests (or Wilcoxon signed-rank tests) will be used. Intergroup comparisons will use independent sample *t*-tests (or Mann–Whitney *U*-tests), and intragroup comparisons before and after treatment will use paired *t*-tests (or Wilcoxon paired signed-rank sum tests). Data obtained by performing repeated measurements at multiple time points will be analyzed using repeated measures ANOVA. Count data will be represented by proportions and rates, with intergroup comparisons of overall efficacy using chi-squared tests (or Fisher's exact test). Intergroup comparisons of proportions will use a 2 × C table chi-squared test. The generalized estimating equation (GEE) model will be used to assess how the primary outcome measures, HAMA, change over time across the two intervention groups. Covariates, such as demographic factors and other relevant variables, will be included in the model to account for potential confounding effects. The GEE model will also be used to examine the effects of the intervention on secondary outcome measures. The GEE model will estimate the average change in outcome measures over time within each intervention group and compare the differences in changes between the intervention and control groups. Interaction terms may be included in the model to examine whether the effects of the intervention vary over time or across different groups of participants. All statistical tests will be two-sided, with a significance level of α =0.05. A *p*-value of < 0.05 will be considered to indicate statistical significance. To better quantify actual changes, missing values will not be inputted. All significance tests will use pairwise missing values, with an alpha value of 0.05.

### 2.18 Ethics and dissemination

If candidates who consent to participate fulfill all inclusion criteria, they will be furnished with an informed consent form. This form provides a comprehensive understanding of the study's scope and potential risks. Participants retain the right to discontinue their involvement at any juncture. Data will be used solely in the aggregate form and will not contain any personally identifiable information. Within the consent form, participants will be prompted to indicate their consent for data usage should they choose to withdraw from the trial. This trial does not involve collecting biological specimens for storage. Moreover, participants will be asked to authorize the research team to share relevant data with personnel from associated regulatory bodies. It is important to note that the trial does not entail the collection of biological samples for storage. This study adheres to the principles of the Helsinki Declaration and has been approved by the Ethics Committee of the Third Affiliated Hospital of Henan University of Chinese Medicine (Approval No. 2023SH-147). The trial protocol has been registered with the Chinese Clinical Trial Registry (ChiCTR2400082221). Results will be disseminated through journal articles, master's theses, or conference presentations.

## 3 Discussion

There is an overlap between the brain networks involved in sleep and emotional regulation (61). Neuroimaging studies have shown that individuals with anxiety exhibit increased activation of the amygdala and insula in function magnetic resonance imaging, which is associated with responses to aversive stimuli and emotional regulation (62). The role of sleep in emotional regulation is based on paradigms of both total and partial sleep deprivation. Individuals exposed to aversive stimuli after total sleep deprivation show a 60% increase in amygdala activation and a threefold increase in the volume of amygdala activation (63). Sleep disturbances have been shown to worsen emotional vulnerability and exacerbate anxiety-related symptoms (9, 64, 65). Sleep deprivation also reduces the activity of the medial prefrontal cortex; increases the activity of limbic system regions such as the amygdala, insula, and anterior cingulate cortex; and decreases the connectivity between the medial prefrontal cortex and the amygdala (66). Sleep deprivation may impair the ability to distinguish between emotional stimuli, disrupting emotional responses and leading to a maladaptive loss of emotional neutrality that is a hallmark of anxiety (67, 68).

Experimental studies have revealed that naturally occurring consecutive sleep loss is associated with day-to-day trajectories of affective and physical wellbeing (69). Consecutive PSD can adversely impact next-day affect, perceived stressors, mindfulness, and cognitive functioning (70). Acupuncture, as a nontoxic, economical, and low-side-effect intervention, has a therapeutic effect on improving cognitive function, but its improvement of GAD with chronic sleep restriction is also worth exploring (59). The purpose of this trial is to observe the brain attention, cognition, and attention bias in the emotional cognitive processing mechanisms of acupuncture's clinical effect on improving GAD with natural PSD.

The inclusion of a healthy participant group in the trial is primarily used to explore the difference in cognitive function between subjects with comorbid GAD and PSD and normal individuals. Subjects and normal individuals will be screened through the Generalized Anxiety Disorder Scale and partial sleep deprivation index, and cognitive functions will be compared by recording EEG data to explore their intrinsic mechanisms. This trial does not set up a drug group and will analyze the indicators 1W and 2W after acupuncture by collecting data on the subjects' brain attention, cognition, and attention bias and conduct a superiority analysis with the sham acupuncture group to explore the clinical effect of acupuncture on improving GAD comorbid with PSD.

This study introduces the concept of naturalistic research to this subject area and observes individuals with comorbid GAD and naturally occurring PSD in a nonclinical sample. The objective is to investigate the biologically close relationship between naturally occurring PSD and the subsequent emergence of anxiety symptoms, as along with the underlying neurocognitive mechanisms in the brain. Applying the principles of naturalistic research to observing the relationship between naturally occurring PSD and negative emotions more closely approximates reality and yields results closer to real-life situations than designing and conducting PSD trials. In clinical observations, we have found that a 2-week intervention can improve the symptoms of sleep deprivation in patients. Choosing a 2-week treatment duration is conducive to further investigating the response time of acupuncture as an early intervention. Considering the long treatment period for GAD, acupuncture, as a low-side-effect treatment method, can aid patients in receiving long-term treatment. When combined with medication therapy, it can enhance treatment effectiveness, making it a recommended alternative therapy. This study focuses on the impact of early adulthood sleep deprivation on generalized anxiety, offering a new perspective for the prevention and treatment of GAD.

Although several measures have been taken to ensure the rigor of the trial, this study has some methodological limitations. The study employs the concept of naturalistic research to observe nonclinical samples with the aim of obtaining results that closely resemble real-life situations. However, due to budget constraints, the sample may not cover individuals from different countries, ethnicities, and races, which could potentially reduce the generalizability of the results. In future research, efforts will be made to expand the sample size to validate the universality of the findings. These limitations notwithstanding, we will use rigorous methods in this study, and we hope that this trial will help provide new insights into acupuncture for the treatment of comorbid GAD and PSD and provide ERP evidence on the execution of brain functions.
